# A Purse-String Traction Technique Using Nylon Loop and Clips for Curative Endoscopic En Bloc Resection of Large Early Gastric Cancer

**DOI:** 10.14309/crj.0000000000002240

**Published:** 2026-07-13

**Authors:** Shuai Tian, Qianqian Chen, Yaoqian Yuan, Jiayan Zhou, Enqiang Linghu

**Affiliations:** 1 Department of Gastroenterology, The First Medical Center, Chinese PLA General Hospital, Beijing, China; 2 Department of Gastroenterology, The 970th Hospital of the PLA Joint Logistic Support Force, Yantai, China

## CASE REPORT

Endoscopic submucosal dissection (ESD) is standard for treating early gastric cancer, but resecting large lesions remains challenging due to poor visibility and insufficient traction.^1,2^ Studies have demonstrated that traction-assisted techniques, such as the pocket-creation method, can achieve a significantly faster median dissection speed (eg, 19.6 mm^2^/min) compared with conventional ESD without traction (15.0 mm^2^/min).^3^ While single-point string traction methods have been reported for colonic ESD by Yamasaki et al., the technique described, a purse-string traction method using a nylon loop and hemoclips, creates a dynamic, multipoint centripetal traction system.^4^ Unlike the single-point, unidirectional linear traction generated by the string-and-clip method of Yamasaki et al, our approach symmetrically retracts the entire lesion circumference through gradual loop tightening, producing a central tent-like effect that is particularly advantageous for large gastric lesions where exposure of the central part of the submucosal plane is critical.^4^ We describe this technique to facilitate curative en bloc resection of large early gastric cancers.

**Figure 1.**
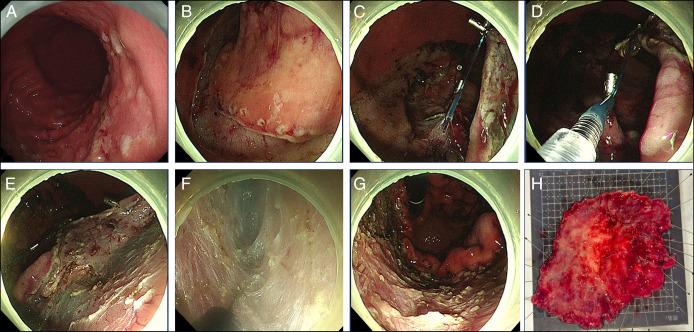
Endoscopic images showing purse-string traction-assisted endoscopic submucosal dissection of a large early gastric cancer. (A) The large early gastric lesion under white light imaging. (B) Status after circumferential mucosal incision and initial submucosal dissection. (C) Placement of several clips anchoring a nylon loop to the lesion margins. (D) The nylon loop is tightened. (E) Providing central retraction and excellent exposure of the submucosal plane. (F) Submucosal dissection proceeding under clear visualization with maintained traction. (G) The wound after lesion removal. (H) The resected specimen, demonstrating en bloc removal.

A 41-year-old woman underwent upper endoscopy for evaluation of occasional acid regurgitation and bitter taste, which incidentally revealed a large superficial gastric carcinoma. The lesion was located on the posterior wall of the mid gastric body, extending from the lesser curvature to the gastric angle, endoscopically estimated at approximately 11 × 10 cm in size. Under white light endoscopy, it appeared as a flat and slightly depressed (Paris IIb + IIc) lesion, with a rough, erythematous mucosa, focal erosion, and thin white coating (Figure 1). Magnifying endoscopy with narrow-band imaging revealed a clear demarcation line with an irregular microvascular pattern (IMVP+) and an irregular microsurface pattern (MS+) according to the Vessel plus Surface classification system. Then an ESD procedure was planned (Video 1). The procedure was performed by a senior endoscopist who performs over 200 ESD procedures annually. After initial marking and mucosal incision with submucosal injection, circumferential dissection began but faced poor exposure (Figure 1). To address this, the purse-string traction technique was applied: several hemoclips (ROCC-D-26-230-C; Micro-Tech, Nanjing, China) were attached to the mucosal edges, and a preloosened nylon loop (Loop40-LD195; LeoMed, Changzhou, China) was anchored to these clips (Figure 1). The total time required from the start of extracorporeal preparation of the nylon loop and hemoclips to the complete endoscopic anchoring and initial tightening of the loop around the lesion margins was approximately 8 minutes. Gradually tightening the loop created stable, tent-like central traction, significantly improving submucosal visualization (Figure 1). It should be noted that once the nylon loop is tightened, its locking mechanism prevents easy loosening. Therefore, a gradual, stepwise tightening strategy was employed, in which the loop was tightened in small increments while continuously monitoring the traction effect and submucosal exposure, and tightening was stopped as soon as a satisfactory operative field was achieved. Dissection then proceeded efficiently (Figure 1). Complete en bloc resection was achieved in 167 minutes from the initiation of lesion marking to complete lesion detachment. After lesion retrieval, prophylactic hemostasis of the wound was meticulously achieved by coagulating all visible and potential bleeding points, including exposed vessel stumps, using hemostatic forceps (FD-410LR; Olympus, Tokyo, Japan) with soft coagulation mode. No hemostatic clips were required for wound hemostasis, and no specific wound closure technique was applied. The wound was left open to heal by secondary intention (Figure 1). The resected specimen was pinned flat on a foam board and photographed with a scale reference. The specimen area was measured using ImageJ software and determined to be 7,581.8 mm^2^ (Figure 1). The dissection speed was calculated as specimen area divided by the marking-to-resection time, corresponding to a dissection speed of 45.4 mm^2^/min. Postoperatively, the patient was managed with fasting, intravenous proton pump inhibitors, and nutritional support. She experienced moderate abdominal pain on postoperative day 1, which gradually resolved with analgesic and symptomatic treatment. No adverse events occurred during the hospital stay. The patient recovered well and was discharged on postoperative day 7.

Final histopathological examination of the en bloc resected specimen confirmed a moderately to poorly differentiated adenocarcinoma of the gastric body, measuring 7.2 × 6.5 cm microscopically. The tumor invaded the submucosa to a depth of 103 μm. All horizontal and deep resection margins were free of carcinoma, and no lymphovascular invasion was identified. The resection was classified as R0 and curative.

The purse-string traction technique using nylon loop and clips establishes a dynamically adjustable, multipoint centripetal traction system by synergistically using hemoclips and a nylon loop. Its core innovation lies in achieving stable lesion lifting and wound gathering through gradual loop tightening, which continuously optimizes the submucosal surgical field for precise dissection. This translates directly into notable clinical benefits: a high dissection speed (45.4 mm^2^/min) enhances efficiency, while maintaining a clear operative plane reduces the risk of muscular injury, bleeding, and perforation, ensuring successful curative en bloc (R0) resection. This case demonstrates the technical feasibility and potential efficiency of the purse-string traction method for achieving curative en bloc resection of a large early gastric cancer. However, as a single case report, the findings should be interpreted with caution and cannot be directly generalized. Comparative studies with conventional ESD and other traction-assisted approaches are needed to further validate the efficacy and clinical advantage of this technique.

## DISCLOSURES

Author contributions: Q. Chen and E. Linghu made contributions to the conception and design of the study. S. Tian and Q. Chen wrote the manuscript. S. Tian, Y. Yuan and J. Zhou edited the manuscript. E. Linghu and Q. Chen corrected the critical revision of the article for important intellectual content. All authors read and approved the final manuscript. E. Linghu is the article guarantor.

Financial disclosure: National Key Research and Development Program of China (2022YFC2503600).

Informed consent was obtained for this case report.
